# Future of the Renal Biopsy: Time to Change the Conventional Modality Using Nanotechnology

**DOI:** 10.1155/2017/6141734

**Published:** 2017-02-19

**Authors:** Hamid Tayebi Khosroshahi, Behzad Abedi, Sabalan Daneshvar, Yashar Sarbaz, Abolhassan Shakeri Bavil

**Affiliations:** ^1^Department of Internal Medicine, Tabriz University of Medical Sciences, Tabriz, Iran; ^2^Medical Bioengineering Department, School of Advanced Medical Sciences, Tabriz University of Medical Sciences, Tabriz, Iran; ^3^Faculty of Electrical and Computer Engineering, University of Tabriz, Tabriz, Iran; ^4^School of Engineering-Emerging Technologies, University of Tabriz, Tabriz, Iran; ^5^Department of Radiology, Tabriz University of Medical Sciences, Tabriz, Iran

## Abstract

At the present time, imaging guided renal biopsy is used to provide diagnoses in most types of primary and secondary renal diseases. It has been claimed that renal biopsy can provide a link between diagnosis of renal disease and its pathological conditions. However, sometimes there is a considerable mismatch between patient renal outcome and pathological findings in renal biopsy. This is the time to address some new diagnostic methods to resolve the insufficiency of conventional percutaneous guided renal biopsy. Nanotechnology is still in its infancy in renal imaging; however, it seems that it is the next step in renal biopsy, providing solutions to the limitations of conventional modalities.

## 1. Introduction

Current renal biopsy methods mostly involve automated biopsy gun and real-time ultrasound-guided technique. The diagnostic accuracy of renal biopsy depends on the different parameters such as experience of the operator, mean number of glomeruli in specimen, and extent of renal involvement [1]. Unfortunately, the conventional renal biopsy method often has several limitations that include the following. (1) Imaging-guided biopsy is an invasive and operator procedure dependent [2]. (2) There is a considerable risk of hematuria and hematoma during and after biopsy procedure [1, 3]. (3) Pathological examination of biopsy specimen is time consuming and can take several days to complete and need expert renal pathologist [4]. (4) Either computed tomography (CT) or ultrasound should be employed for guiding the biopsy needle towards the kidney using real-time image acquisition. CT-scan can determine the exact position of the biopsy needle in relation to the renal position, but this imaging technique exposes the patients to the considerable radiation. In contrast, ultrasound guidance is a nonionizing technique for needle guiding; however, some problems like poor needle visibility, especially in obesity patients, has limited its use [2]. (5) Kidney biopsy is associated with local pain in needle penetration site and renal capsule. (6) It is important to determine the optimal size of needle due to its effect on specimen size. The mean diameter of a typical glomerulus is 100 to 250 *μ*m. Therefore, the needles with diameter more than 600 um can collect high numbers of glomeruli [KIM], while increasing the renal biopsy complications such as hematuria and merely leading to the renal loss [5]. In contrast, the needles with diameter less than 400 *μ*m have a fewer side effects, while are unable to collect a sufficient number of glomerulus and the collected ones are usually fragmented or lost [6]. (7) In practice, usually more than one biopsy pass is required to obtain the desired result that increase the risk of biopsy [1]. (8) The recovery phases of renal biopsy consist of an inpatient period of 1 to 3 days and the outpatient period of about one week [3]. (9) Usually, renal involvement is not homogeneous; therefore, small specimen cannot represent the real condition of kidney and conventional renal biopsy method is not always a reliable indicator for the overall condition of the kidney [7]. As described, renal biopsy is an invasive method and its use is limited by low diagnostic accuracy due to local evaluation of the renal parenchyma. Small number of glomeruli cannot be the representative of one million nephrons. In other words, it seems that making a medical decision based on renal biopsy results may be misleading and can result in over- or undertreatment of the patients. It has been established that “~*12% of the biopsies did not shed light on the diagnosis and were unhelpful in patient management, another *~*11% were nondiagnostic, and an additional 1.5% failed to yield enough tissue for examination*” [8]. In the recent years, several different practical techniques have been proposed to improve the efficiency of renal biopsy by using different guiding techniques and high-performance needles. The main milestones of the renal biopsy are summarized in Table 1.

This is the time to address some new diagnostic methods to resolve the insufficiency of conventional percutaneous guided renal biopsy. Several techniques have been proposed for improving the needles visibility in ultrasound-guided biopsy by increasing their echogenicity. These techniques include the use of coating agents, dimpling the needle tip, and texturing with different methods [13]. This improvement could aid accurate localizing of the needle and decrease the number of biopsy passes in order to reduce some renal biopsy complications. Despite of these advancements, as described earlier, conventional renal biopsy suffers from serious limitations. As a result, in the last decades several attempts have been made to enable clinicians to assess renal structure and function in details and to avoid the need to collect renal specimens. This can be partially accomplished either through employing the modified version of conventional imaging technologies such as pulsed ultrasonography (US), micromagnetic resonance imaging (*μ*-MRI) and micro-CT (*μ*-CT) or through using multimodal approaches such as single-photon emission/CT (SPECT/CT), positron emission tomography/CT (PET/CT), PET/MRI, and PET/optical [14]. In addition, development of different imaging contrast agents improved the diagnosis accuracy of medical imaging techniques [15].  Table 2 shows the main properties of the known medical imaging modalities including the spatial/temporal resolution of technique, operator dependency, and potential hazards to the subjects (e.g., radiation).

### 1.1. High-Performance US

**Figure 1:**
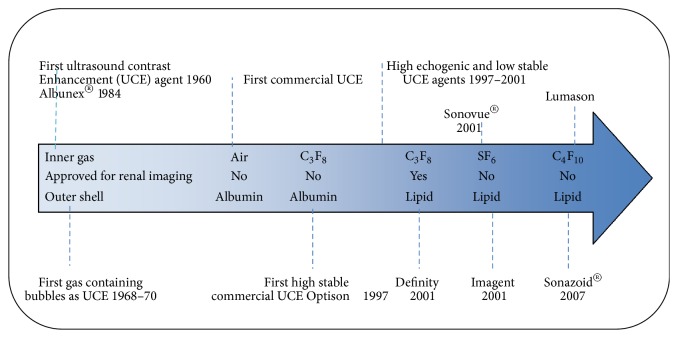
The timeline of development of US-based contrast agents.

The image quality of ultrasonography has dramatically improved in the last decades, but it has been proven that “*commercial ultrasound systems lack sufficient resolution to differentiate exactly between tissue planes*” [21]. The resolution of US depends on the frequency of the transducer, “*frequencies between 30 MHz and 50 MHz provide resolution to between 100 μm and 60 μm respectively*” [21]. In the recent years, high frequency transducer has been introduced for pushing the resolution and contrast limits of conventional ultrasonography. This method can provide an imaging depth of 50 mm with a spatial resolution down to 70 *μ*m [22, 23]. The high frequency transducer can be used as a powerful tool for diagnosing of undetectable lesions such as early detection of prostate cancer risk in rats [24]. Unfortunately, the penetration depth of high frequencies transducer is limited, and deep organs cannot be studied in their entirety [25]. To data, several studies have confirmed the efficiency of renal contrast-enhanced ultrasonography (CEUS), in early diagnosing of renal involvement [26–32]. However, CEUS is still used infrequently in clinical practice [33, 34]. In this method, to increase the contrast of ultrasound imaging in clinical practice, shelled, gas-filled microbubbles are routinely injected intravenously to increase the mismatch in acoustic impedance between tissues and thus help detect and characterize focal lesions. Indeed, the first FDA approved contrast agent in clinical use is the Gd^3+^ DTPA chelate [35]. The first FDA approved contrast agent used in the US was Albunex (since discontinued) in 1994, which was only approved for echocardiology [36]. The timeline of development of US-based contrast agents has been shown in Figure 1. US contrast agents usually are air or gas filled microbubbles or microspheres that are albumin, lipid, or polymer coated [37]. In our knowledge, Definity®, a C3F8-filled and lipid-coated microbubble, is the first FDA-approved contrast agent for renal imaging. Ultrasound contrast agents can be divided into five different classes: (1) nonencapsulated gas microbubbles, (2) stabilized gas microbubbles, (3) encapsulated gas microbubbles, (4) microparticle suspensions or emulsions, and (5) gastrointestinal.

**Figure 2:**
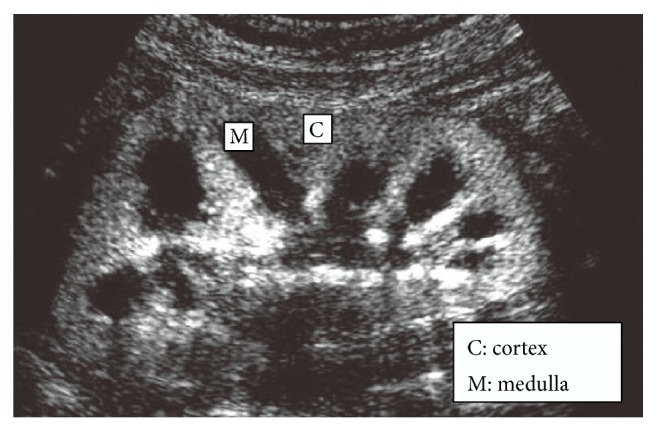
Improving the echogenicity of renal vascular employing MPs.

Tsuruoka et al. claimed that Sonazoid™ can be used as a safe efficient contrast agent in evaluation of dynamics of renal microcirculation and early diagnosing of CKD. These microbubles can improve the visualization of renal vascular by improving the echogenicity of flowing blood [38], Figure 2. These microbubbles usually have diameter between 1 and 8 *μ*m [39]. As a result these particles could not be excreted with kidney filtration. This property will help physicians in better visualization of renal microvasculature and early diagnosing of microvasculature diseases such as small vessel vasculitis and thrombotic microangiopathy. As seen in Figure 1, all of the newly developed microbubbles are lipid coated. Besides improving the general contrast of the imaging system, an ideal contrast agent needs to provide additional information about molecular and cellular content of lesion. Currently, researchers have begun to focus on the development of contrast agents with specific binding capabilities [40]. Infiltration of leukocytes in kidney parenchyma can be a potential detector for type of renal involvement. It has been established that microbubble-leukocyte interactions depend on microbubble shell composition [41]. Lipid-coated microbubbles can interact with activated leukocytes better than albumin-coated ones [42]. In our experience, this property is not enough in its own right, since an ideal sonographic contrast agent should have low affinity to tubular epithelial cells and Mesangial cells.

### 1.2. High-Performance CT

CT is a high-resolution method, which can characterize the mass as solid or cystic [43]. At the present time, CT is one of the most commonly used diagnostic tools in renal imaging. This method enjoys the advantage of having higher penetration depth compared to the US and, on the other hand, has higher resolution [44]. Unfortunately, CT has limited utility in imaging soft tissues such as fat/muscle, normal organ/tumors, or cortex/medulla because of similar X-ray absorption among low-density structures [20]. As a result, the contrast of CT-based images will be very low making the interpretation of the image difficult [45]. In the last decades, several attempts have been made to push the limits of CT and it seems that “*its potential is only starting to be explored*” [46]. High-resolution X-ray Computed Tomography (HRXCT) or microcomputed tomography (*μ*-CT) is one of the several X ray-based imaging technologies [47] which can be considered as a potential imaging modality to overcome the limitations of conventional CT. The *μ*-CT permits noninvasive examination of gastrointestinal tract, cardiovascular system, renal tract, liver, lungs, bone, cartilage, tumorous tissue, and so forth [48]. It has been shown that *μ*CT can be used for accurate studying of macro-to-microvascular changes during early-to-late-stage progressive renal involvement [44]. It seems the main drawback of this technique is that its resolution depends on the scan time; “*it took up to 30 min to scan single maize kernels at a resolution of 13.4 mm, whereas a two hour scan time was needed to obtain a 6 mm resolution*” [46]. However, in medical imaging long scan time can be problematic due to the following reasons: (1) the patients will be exposed to the more dangerous high dose ionizing radiation for long time and (2) minimal movement of the subject during the imaging procedure will cause motion-blurring artifacts [49]. As a result, some patients may need anesthesia for a *μ*-CT scan, but anesthesia is not always possible. In addition, formation of destination image can take several hours because of large data volumes. Nano-CT has been developed in order to compensate the deficiencies of the conventional *μ*-CT [50]. Scanning time has been decreased, samples sizes increased, and resolution improved in nano-CT compared to *μ*-CT [50]. In spite of these developments, none of these techniques is ideal for monitoring ultrastructure of renal parenchyma in itself because of the intrinsic and/or technical problems. In the recent years, several efficient exogenous CT contrast agents have been introduced for pushing back the limitations of the previously described CT-based methods. These agents usually are small iodinated molecules and barium sulfate suspensions [51]. None of these are optimal [52]. Contrast-related acute renal injury (CI-AKI) is a serious restriction in using from small iodinated as imaging contrast agents [53]. Barium sulfate suspensions are employed only for upper gastrointestinal X-ray examinations [54]. Barium sulfate suspensions are rapid renal excretion and also known to be renal-toxic [52]. In addition iodinated agents suffer from short blood-circulation time, nonspecificity in in vivo imaging, and low contrast efficiency [52]. In the ideal situation, a large dose at a high rate would be optimal. This, however, must be weighed against safety, practicality, and cost. In the last decades, NPs have attracted great interest in bioimaging due to unique electronic, magnetic, optic, catalytic, and thermodynamic characteristics [55]. It has been established that the efficiency of each NP in CT imaging is dependent on its density and atomic number [48]. The relationship [56] between density (*ρ*), atomic number (*Z*), atomic mass (*A*), X-ray energy (*E*), and X-ray absorption coefficient (*μ*) of each NP has been shown in

(1) $$\begin{array}{c} \mu \approx \frac{\rho Z^{4}}{AE^{3}}. \end{array}$$

As it is clear, *μ* strongly depends on *Z* [56]. In other words, NPs with high atomic number will produce more brighter signal on CT images. However, an ideal contrast agent should show high specificity toward target tissue for increasing the *μ* difference between the target tissue and surrounding tissue. Fortunately, the surface of these NPs can be modified with peptides, proteins, and antibodies, which make these NPs target-specific. NPs containing Au, Bi, Ta, Yb, and so forth can satisfy both of these requirements. Due to high *Z*, metallic NPs can provide better contrast compared to iodine NPs. Currently, research efforts are focused on the development of contrast agents that possess specific binding capabilities [40].

### 1.3. High Resolution MRI

MRI has the highest soft tissue contrast resolution of the imaging between all methods in use today [57]. Although MRI provides good anatomical information with appropriate resolution, it suffers from low sensitivity and temporal resolution. MRI inaccuracy results from different artifacts such as incomplete fat suppression, air bubbles in the bloodstream, and calcification [58]. This drawback can be overcome by combining MRI with other sensitive imaging modalities, such as PET, SPECT, and optical imaging [59–61]. In addition, fast MRI has been improved the spatial and temporal resolution of MRI modality, which enables the investigation of renal in detail. Ultra-high field MRI (UHF-MRI) is one of the several MRI-based imaging technologies which has been introduced as a potential technique to realize renal ideal imaging modality. Irazabal et al. employed ultra-high field MRI for assessment of polycystic kidney disease (PKD) in small rodent models of PKD [62]. One of the main advantages of the UHF-MRI is its use as a potential tool for assisting the experts to early diagnose some advanced kidney diseases such as medullary spongy kidney, medullary cystic disease, kidney malignancies in situ, and autosomal dominant polycystic kidney disease (ADPKD) [62]. ADPKD is the most common renal genetic disorder, which may not be diagnosed until ages <20 years using the conventional imaging modalities such as CT and US. In another example, conventional imaging modalities such as, CT, MRI, and PET cannot distinguish metastatic LN involvement, because of poor sensitivity and/or specificity and the inherent limits on size of nodal metastases that can be detected [63]. It seems UHFMRI can be employed as a powerful tool for the accurate diagnosis of ADPKD disease in early stages. However, UHFMRI cannot provide detailed information about the renal diseases in cellular levels and “*another much-discussed aspect of ultra-high field imaging that has been put forward as a possible obstacle to clinical use are physiological side-effects of the magnetic field*” [64]. In the recent years, several attempts have been made to compensate the drawbacks of UHFMRI. Despite these efforts, Saito et al. claimed that the image ex vivo resolution of *μ*-CT is higher than that of UHFMRI [65]. In addition, scanning time of *μ*-CT scanners is shorter than UHFMRI scanning systems [65]. These findings are in line with other studies; “*several studies which compare the performance of X-ray μ-CT against other imaging techniques, that is, MRI, has revealed that X-ray is less costly and more convenient*” [46]. As explained before, in comparison to the other imaging techniques, the main advantage of MRI is its excellent spatial resolution, whereas it suffers from the limited sensitivity [66]. In a simple word, conventional MRI can detect large lesions easily while it cannot detect smaller lesions due to low SNR [67]. SNR can be defined as the ratio of desired signal power to the background noise power [68]. As the size of lesions decreased, the SNR will be decreased due to low power desired signals. Magnetic nanoparticles (MNPs) have been proposed as a potential candidate for overcoming this limitation by increasing SNR. Fortunately, in the last decades, several MRI contrast agents have been introduced for improving the sensitivity of this modality. Magnavist is the first FDA approved contrast media for MRI [35]. These agents can improve the sensitivity of MRI in the early diagnosing of renal involvement [69].

As shown in Table 3, most of the FDA-approved contrast agents for MRI are gadolinium based [70]. Gadolinium itself is toxic and should be coated with other chemicals [71]. This property will help the researchers on the development of contrast agents with specific binding capabilities. Gadolinium is highly paramagnetic substance [72]. Shokrollahi divided the MRI contrast agents into two categories paramagnetic compounds, including lanthanides like gadolinium, and super-paramagnetic magnetic nanoparticles such as iron oxides [66]. Unfortunately, gadolinium based contrast agents cannot be used in patients with low glomerular filtration rate (GFR) because of the risk of NSF [70]. Iron oxide MNPs are FDA-approved contrast that are nontoxic at a low dose [73]. It has been proven that NP-based MRI can be used as a powerful tool in diagnosing acute renal failure (ARF) [74] before the serum creatinine even begins to rise [75]. Gadolinium based contrast media are classified as T1 agents, while ferromagnetic large iron oxide media are known as T2 agents [76]. SPIOs can penetrate cells. From the standpoint of clinical diagnosis and cellular imaging, the image contrast produced by such agents is far less desirable than that by the T1 agents. Magnetic particle imaging (MPI), an emerging tomographic imaging method, directly measures the magnetization of iron oxide nanoparticle tracers. The MPI signals derived from the nonlinear remagnetization response of super paramagnetic iron oxide nanoparticles (SPIONs) to an oscillating magnetic field. Efforts to propel MPI forward as an imaging method by improving its spatial resolution, imaging speed, and sensitivity have expanded [16]. Magnetic particle spectroscopy (MPS) has been developed in parallel with the reconstruction of the MPI scanners to allow researchers to evaluate, characterize, and optimize the properties of tracers at a faster pace and lower costs, independent of the confounding complexities of the hardware and software technologies of a 3D MPI scanner.

### 1.4. Optical Imaging

Optical imaging is a potential technique, which allows physiological and pathological activities to be studied in vivo. This modality has been introduced as a potential tool for studying the renal involvement in cellular level [102]. This method is widely used because it is both high performance and cost effective [103]. Recent development in optical imaging offers a myriad of procedures, which are useful for studying the structure and function of different organs such as kidney, brain, and colon [104]. For example, multiphoton microscopy (MPM) can provid real-time movies of the renal function in vivo without damaging tissue [105]. Despite of recent developments, optical imaging modalities suffer from some limitations such as low penetration depth. The process that limits the imaging depth and contrast of NIR imaging is scattering rather than absorption. In addition, the contrast resolution of modalities will be degraded at higher depth due to light scattering [106, 107]. In the recent years, several procedures have been proposed to overcome the penetration depth limitation of optical imaging. Wang et al. claimed that both the depth and the contrast optical imaging can be enhanced by the application of agents [108]. Higher concentration of agents causes more water loss of skin tissue and a stronger optical clearing effect. Taruttis et al. claimed that this problem can “*be overcome by adding ultrasound detection to optical excitation in exploitation of the photoacoustic effect*” [106]. Photoacoustic (PA) combines the high resolution of US with the high contrast of optical imaging techniques. PA can be used for molecular visualization of kidney due to its high tissue penetration and appropriate spatial resolution [100]. It has been established that endogenous contrast agents (hemoglobin and melanin) can improve the spatial resolution of PA modality in deep tissues. However, in some cases, such as solid tumors and lymph nodes, endogenous contrast agents are not available, and exogenous contrast agents should be used to overcome this problem. It has been established that CNPs based PA can provide high resolution images from in depth organs with adequate contrast. Besides this feature, GNPs also have high scattering cross-section in the red region of the spectrum. This property is crucial for development of contrast agents for optical imaging in living organisms because of light penetration depth in SPIE. Natural nanoparticles are also widely used in PA, but they have small size (<2 nm) and can distribute to a wide range of tissue nonspecifically. As a result, these agents cannot provide sufficient imaging contrast in the region of interest against surrounding tissue [109]. In the last decades, carbon-based nanocomposites are extensively used in PA imaging. These NPs are strong NIR absorbance and in contrast to gold NPs carbon-based NPs are nontoxic and photostable. Based on the narrative summarized above, optical imaging is still in its infancy and it seems that optical biopsy is the next step in medical imaging, providing solutions to the limitations of conventional modalities [110]. The advantages and disadvantages of small numbers of available internal imaging modalities have been summarized in Table 4.

## 2. Future Prospect: An Ideal Renal Imaging Modality

As described in earlier sections, despite of the recent significant advances in medical imaging technology, there are still certain applications for which the conventional imaging modalities are not the suitable solution and more developments are needed in the contrast, resolution, and the penetration depth in the future [137]. Kidney is one of the key organs, which plays an important role in regulating various physiologic mechanisms. None of the conventional imaging modalities are consistently effective in early diagnosing of renal involvement. The kidney generally lies 5 to 10 cm under the skin surface in nonobese people. With ease of access, the kidney is an ideal organ in which low penetration imaging modalities can be applied successfully. For researchers, the ideal imaging technique should have the spatial resolution of MRI, temporal resolution of ultrasound, and the sensitivity of PET [16]. It can be claimed that an ideal noninvasive renal imaging technique should have the following properties [138]: (1) it should be a safe, nontoxic, and low ionization imaging technique; (2) be able to obtain dynamic images with high resolution and rich contrast for assessment of single nephron function; (3) be able to provide centimeters penetration depth into biological tissue; and (4) be of acceptable cost and offer 3D fast-imaging in living subjects. In the recent decades, several attempts have been done for realization of ideal diagnostic imaging modality by promoting the MRI, CT, and US imaging techniques. However, because of the technical complexity and/or intrinsic limitations, most of the efforts were limited to the construction of advanced animals living imaging tools [65]. Fortunately, in the recent years several methods have been proposed for improving the acquisition time of new optical imaging modalities [139]. The safety is one of the primary requirements of any imaging modality. Optical, acoustic, magnetic, and low-exposure X-ray based imaging modalities are safe when compared to other techniques such as multiexposure X-ray and nuclear medicine based imaging. Nevertheless, in spite of safety of these modalities, low imaging depth of penetration and/or poor resolution limits their applications. Theoretically, there is a tradeoff between imaging depth of penetration and spatial resolution in mechanical [140] or electromagnetic [141] based imaging modalities. In other words, the resolution of optical imaging modalities will be degraded at higher depth due to light scattering [106, 107]. Therefore, beside high spatial resolution, an ideal imaging modality should have the sufficient imaging penetration depth in tissue. It seems understanding the wavelength, refractive index, Brownian motion, orientation, and size and phase function of tissue scatters can result in modern powerful medical imaging modality [120]. The penetration depth of ~10 centimeters allows complete investigation of organs such as liver, pancreas, and kidney in nonobese ones. This can be achieved in five ways. (1) It seems improving the conventional imaging technologies can help provide better understanding of internal organs. (2) Using semi-invasive methods such as optical biopsy [142], intraoperative laparoscopic ultrasound (usually 4–20 MHz), rigid or flexible probe, [143, 144], microultrasound probe [24], endoluminal US (usually 12–40 MHz) [145], and so forth can be helpful in accurate evaluation of internal organs such as kidney. As cleared earlier, renal involvement is not homogeneous. Therefore, some regions within the kidney can be identified as suspicious for renal involvement based on this technique and biopsy can be done to these regions. (3) The development of hybrid imaging methods such as optic/ultrasound provides greater imaging depth penetration in biological tissues and allows obtaining high resolution 3D images from internal organs [8, 146]. (4) In our knowledge, depth of penetration can be considered as a common limiting factor in almost all of high resolution mechanical and electromagnetic based imaging modalities. Metamaterial lenses makes the internal organs appear closer than they actually are. This method makes in-depth imaging possible, while maintaining the high depth-to-resolution ratio. The resolution of this technique in tissue imaging was three times better than diffraction limits [147]. (5) Development of new computer algorithms will definitely help to improve the performances of different medical imaging modalities in the future [148].

**Figure 3:**
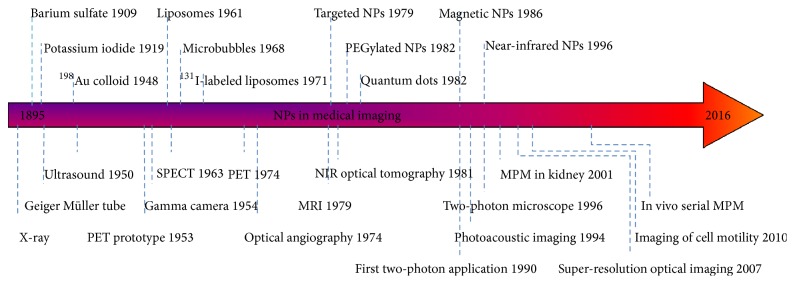
Development of NPs as medical imaging contrast agents over the past decade.

As cleared, despite recent significant advances in medical imaging technologies, nonmodality has an optimal resolution and penetration depth. Despite of continuous development, most of the medical imaging theories are a vision of the future and considerable effort should be dedicated to make them true. The purpose of this paper is to introduce a strategy to guide current and future activities to achieve this vision. We hypothesise that one of the potential application areas of future imaging technologies could be in assessing kidney where imaging depth of ~10 centimeter is sufficient to evaluate early renal involvement. Based on these evidences, it can be predicted that a safe high-resolution depth imaging modalities can potentially be employed for the monitoring and diagnosing of early renal involvement [110]. This technology will decrease the need for renal biopsy and as a result can remove any side effects of the conventional renal biopsy and enabling overcoming the limitation of the conventional methods. Achieving this target will involve several phases and may require new procedures. However, any improvement in this area will benefit the performances of the conventional medical imaging modalities. The first benefit of this new nonionization modality will be the medium-resolution high depth capability to determine the exact position of the biopsy needle in relation to the renal position, allowing for minimization of the number of biopsy passes and consequently reducing the recovery period. Moreover, high-resolution medium depth modality will allow complete and accurate investigation of internal organs such as transplant kidney, liver, and pancreas in nonobese ones. Finally, a high-resolution high depth imaging modality can make a revolution in renal disease study. Advanced imaging techniques are just one of the several methods that can be used for early diagnosis of kidney involvement. It has been claimed that accurate analysis of salivary urea can be used as a proper tool in diagnosing of chronic kidney disease [149]. In addition, development of different imaging contrast agents improved the diagnosis accuracy of medical imaging techniques [15]. It can be claimed that the key to further developing the convectional medical imaging modalities, as well as developing entirely new methods, lies in the use of contrast agents. Magnetic particle imaging (MPI) is an new modality in biomedicine that is designed to image the amount and location of super paramagnetic nanoparticles in animals or humans with high spatial and temporal resolution [150]. MPI's decreased image acquisition times foster the making of tomographic images with high spatial and temporal resolution. In addition, the contrast and sensitivity of MPI are improved significantly in compared to other convectional medical imaging modalities, such as MRI, X-ray scans, US, CT, PET, and SPECT [15]. The timeline of development of contrast agents as medical imaging media has been shown in Figure 3.

## Figures and Tables

**Table 1:** The main milestones of the renal biopsy in last century.

Stage	Year	Reference(s)
First renal biopsy	1901	[2]
First radiography-guided percutaneous renal biopsies	1944	[2]
Cutting needle	1954	[9]
Percutaneous renal biopsy under direct radiology control	1962	[10]
Ultrasonic localization for renal biopsy	1974	[11]
Using the automated biopsy gun with real-time ultrasound for native renal biopsy	1979	[12]
Spring-loaded, automated, cutting-needle biopsy	1980s	[1]

**Table 2:** Comparing the performances of different imaging modalities.

	Sensitivity	Spatial resolution	Temporal resolution	Acquisition times speed	Soft tissues contrast	Radiation exposure	General anesthesia	Operator dependence	Signal used
MRI	Low [16]	25–100 *μ*m	Minutes to hours	Very low	High [17]	No [18]	Yes [19]	No	Radio waves
CT	Low [16]	50–200 *μ*m	Minutes	Medium	Low [20]	Yes	Yes	No	X-ray
US		~1 mm	Second	High	High	No	No	Yes	Mechanical wave
PET	High [16]	1-2 mm	10 seconds to minutes	Medium		Yes	No		*β*/*γ*
SPECT	High [16]	1-2 mm	Minutes	Low		Yes	No		Γ

**Table 3:** Different contrast agents.

Medical imaging types	Modality	FDA approved contrast agents	Particle size	Comment(s)
Structural	MRI	AMI-25 (Feridex™) [77]	∼58 nm [78]	T_2_-agent
		Schering (Resovist®) [79]	~21–46 nm [80]	
		OMP50 [81]	~300 nm [82]	
		Feridex (FDA cleared) [71]	~300 nm [82]	
		Ferumoxytol [83]	~300 nm [82]	Can be used in patients with CKD stages I–V or ESD
		AMI-121 (Ferumoxsil™) [81]	~300 nm [84]	
		Gadolinium contrast agents [85]		
		Gadodiamide (Omniscan™) [86]		Linear nonionic, high nephrogenic systemic sclerosis (NSF) risk
		Gadobenate (Multihance®) [86]		Linear ionic
		Gadopentetate (Magnavist®) [86]		Linear ionic, high NSF risk
		Gadoteridol (ProHance®) [86]		Macrocyclic ionic
		Gadofosveset (Ablavar®) [86]		Linear ionic
		Gadoversetamide (OptiMark™) [86]		Linear nonionic
		Gadobutrol (Gadovist®) [86]		Macrocyclic ionic
		Gadoterate (Dotarem®) [87]		Macrocyclic ionic
		Gadoxetate (Primovist®) [87]		Linear ionic
		Iron oxide MNPs [73]		
		Super paramagnetic iron oxides (SPIO) [88]		
		Omniscan (FDA cleared) [71]		
		3He (under investigation) [89]		
		Manganese dipyridoxaldiphosphate (Mn-DPDP) [90]		
		MnCl_2_ [91]		

Structural	CT	Iopromide (Ultravist®) [92]	~200 nm [93]	
		Iopamidol (Isovue 370) [94]		Nonionic monomers
		Iohexol (Omnipaque 350) [95]		Nonionic monomers
		Gold nanoparticles		

Structural	US	Albunex [36]	1–8 *μ*m [39]	
		Optison® [96]		
		Lumason® [96]		
		Definity [97]		
		Imagent® (formerly Imavist™) [98]		

Structural	Multiphoton microscopy (MPM)	Nanotubes [99]		

Structural	OCT	To date, there are no FDA-approved contrast agents for imaging with OCT.		

Structural	Photo acoustic imaging [100]	Clofazimine (CFZ)		

Functional	PET	Compound of ^18^F and natural nanoparticles (lipoproteins, viruses and ferritin) [101]		
	fMRI	Gd-DTPA		

Spectral	Fluoresce	Indocyanine green		
		Fluorescein		
		Agent methylene blue		
		Demeclocycline		

Spectral	Near infrared absorption spectroscopy	Indo Cyanine Green (ICG)		

Spectral	Hyperspectral imaging			

**Table 4:** Advantages and disadvantages of small numbers of available imaging modalities.

Method	Some advantages	Some disadvantages	Readiness for clinical use [8]	Acquisition time	Penetration depth	Resolution	Integrating capacity with conventional endoscopes
Photo acoustic microscopy	(1) It can provide deep, high resolution optical images of internal organs [107]. (2) Minimize the motion artifacts [106]. (3) Provide not only anatomical/structural but also functional and molecular contrast [107]. (4) It has the capability of two- and three-dimensional reconstruction of the acquired images [111].	(1) It is unable to image dynamic processes in living tissue [112].	Yes	Several minutes [112]	2–5 cm [107, 113]	Axial resolution of 15 *μ*m [114] Lateral resolution of 45 *μ*m [114]	Yes [115]

Optical coherence tomographic (OCT)	(1) It can enables high resolution depth imaging using low coherence interferometry [116]. (2) It has the capability of two- and three-dimensional reconstruction of the acquired images [117]. (3) It can function as a type of “optical biopsy” [5, 118]. (4) OCT can distinguish tissue types in kidney based on attenuation coefficient [119]. (5) Polarization-sensitive OCT has deeper penetration depth compared to OCT [120].	(1) It cannot provide imaging of deep tissue [5].	Yes	1–3 seconds [121, 122]	1 to 2 mm [117]	Axial resolutions of 1–10 *μ*m [118]	Yes [118, 123]

Raman spectroscopy	(1) Integration with OCT; Raman spectroscopy provides an objective histopathological diagnosis [120].	(1) It is sensitive to tissue movement [120].	Yes	Several minutes [120]	Several millimeters [124]	Undefined resolution [124]	Yes [125]

Ultrasound biomicroscopy (UBM)	(1) It is actually better suited to whole embryo imaging than OCT [126]. (2) Ultrasound biomicroscopy has a resolution 5 to 10 times that of a 10-MHz ultrasound probe [127].	(1) Low resolution [126].	Yes	Acquire real time images [128].	~5 mm [129].	In vivo lateral resolution of 50 *μ*m [129]. In vivo axial approaching 25 *μ*m [129]	Yes [129]

Confocal laser scanning microscopy (confocal microscopy)	(1) In vivo three-dimensional imaging [130]. (2) It can record multiple sectional images of microobjects via their depth direction [131].	(1) Confocal microscopy cannot replace UBM in making specific diagnosis [132].	Yes	Few seconds [133]	Few hundred microns [126]	~0.2 *μ*m [134]	Yes [8]

Narrow band imaging (NBI)	(1) NBI can improve visualization of tumors and vessels [135]. (2) There is no need for an intravesical dye [135].	(1) Limited view [136]	Yes	Several minutes [136]	Blue 170 *μ*m Green 240 *μ*m [104]	High [135]	Yes [104]
